# Identification of 1*H*-pyrazolo[3,4-b]pyridine derivatives as potent ALK-L1196M inhibitors

**DOI:** 10.1080/14756366.2019.1639694

**Published:** 2019-08-11

**Authors:** Yunju Nam, Dongkeun Hwang, Namdoo Kim, Hong-Seog Seo, Khalid B. Selim, Taebo Sim

**Affiliations:** a KU-KIST Graduate School of Converging Science and Technology, Korea University, Seoul, Republic of Korea;; b NDBio Therapeutics Inc., Incheon, Republic of Korea;; c Cardiovascular Center, Korea University Guro Hospital, Seoul, Republic of Korea;; d Chemical Kinomics Research Center, Korea Institute of Science and Technology (KIST), Seoul, Republic of Korea;; e Department of Pharmaceutical Organic Chemistry, Mansoura University, Mansoura, Egypt

**Keywords:** Anaplastic lymphoma kinase, ALK-L1196M mutant, pyrazolopyridine-based inhibitor

## Abstract

Anaplastic lymphoma kinase (ALK) has been recognised as a promising molecular target of targeted therapy for NSCLC. We performed SAR study of pyrazolo[3,4-b]pyridines to override crizotinib resistance caused by ALK-L1196M mutation and identified a novel and potent L1196M inhibitor, **10g**. **10g** displayed exceptional enzymatic activities (<0.5 nM of IC_50_) against ALK-L1196M as well as against ALK-wt. In addition, **10g** is an extremely potent inhibitor of ROS1 (<0.5 nM of IC_50_) and displays excellent selectivity over c-Met. Moreover, **10g** strongly suppresses proliferation of ALK-L1196M-Ba/F3 and H2228 cells harbouring EML4-ALK via apoptosis and the ALK signalling blockade. The results of molecular docking studies reveal that, in contrast to crizotinib, **10g** engages in a favourable interaction with M1196 in the kinase domain of ALK-L1196M and hydrogen bonding with K1150 and E1210. This SAR study has provided a useful insight into the design of novel and potent inhibitors against ALK gatekeeper mutant.

## Introduction

**Figure 1.**
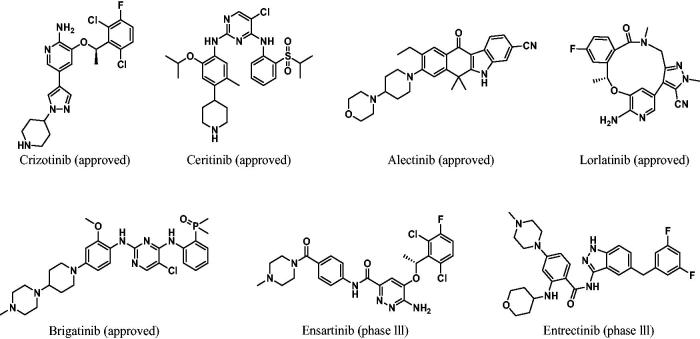
ALK inhibitors on market or under clinical trials.

Anaplastic lymphoma kinase (ALK), a member of the insulin receptor tyrosine kinase superfamily, was originally identified as NPM (nucleophosmin)-ALK fusion protein generated by (2;5)(p23:q35) chromosomal translocation in anaplastic large cell lymphoma (ALCL)^1^^,^^2^. ALK gene rearrangements result in various ALK fusion oncogenes including EML4 (echinoderm microtubule-associated protein like 4)–ALK^3^ in lung cancers, TPM3 (tropomyosin 3)-ALK in ALCL^4^ and TPM3/4–ALK^5^ in inflammatory myofibroblastic tumours (IMT)^6^. ALK gene aberrations found in several human cancers include rearrangement, activating point mutations^7^ such as F1174L/R1275Q, and gene amplification. ALK has received a great deal of attention as a promising therapeutic target for targeted cancer therapy and, as a result, enormous efforts have been devoted to developing ALK inhibitors. While various scaffolds such as pyrimidine, aminopyridine^8^^,^^9^, benzo[b]carbazolone^10^, indazole^11^ have been exploited to identify new ALK inhibitors, 2,4-diarylamino pyrimidines^2^^,^^12–21^ have been the most common molecular platform for discovering ALK inhibitors including ceritinib and brigatinib. Crizotinib^22^^,^^23^ was approved in 2011 as an ALK inhibitor for treatment of NSCLC patients harbouring EML4-ALK fusion oncogene. This substance was also approved in 2016 for treatment of ROS1-positive NSCLC. In 2018, crizotinib was given a breakthrough therapy designation to treat ALK-positive relapsed/refractory anaplastic large cell lymphoma (ALCL)^24–27^ patients. However, acquired secondary mutations (L1196M, G1269A, F1174L, S1206Y, 1151 T-ins, L1152R, C1156Y and G1202R) occurring in the ALK kinase domain resulted in resistance to crizotinib^18^^,^^20^^,^^28–30^. The ALK gatekeeper mutation L1196M is the most frequent secondary mutation taking place in NSCLC patients and, consequently, a significant effort has been made to identify novel and potent L1196M inhibitors^31^. These efforts have led to the development of second-generation ALK inhibitors including ceritinib^32^^,^^33^, alectinib^10^, lorlatinib^8–9^, brigatinib^34^^,^^35^, ensartinib^36^ and entrectinib^11^ that circumvent the acquired secondary mutations^21^^,^^37^ (Figure 1).

As part of continuing studies aimed at discovering structurally distinct and potent ALK-L1196M inhibitors, we carried out SAR investigation using pyrazolopyridine derivatives bearing 3-fluorophenyl sulfone moiety. The substances utilised in this study were designed based on the reported 3-amino-5-substituted indazole-based ALK inhibitors including entrectinib^11^^,^^38^. SAR exploration using novel pyrazolopyridine derivatives led to identification of **10 g** as a novel and potent inhibitor against ALK-L1196M (IC_50_ < 0.5 nM) as well as against ALK-wt (IC_50_ < 0.5 nM). It is worthwhile noting that entrectinib^11^^,^^38^ inhibits ALK-wt with an IC_50_ value of 12 nM.

## Experimental

### Chemistry

Unless otherwise described, all commercial reagents and solvents were purchased from commercial suppliers and used without further purification. All reactions were performed under N_2_ atmosphere in flame-dried glassware. Reactions were monitored by TLC with 0.25 mm E. Merck precoated silica gel plates (60 F254). Reaction progress was monitored by TLC analysis using a UV lamp, ninhydrin, or *p*-anisaldehyde stain for detection purposes. All solvents were purified by standard techniques. Purification of reaction products was carried out by silica gel column chromatography using Kieselgel 60 Art. 9385 (230 − 400 mesh). The purities of all compounds were shown to be over 95% by using Waters LCMS system (Waters 2998 photodiode array detector, a Waters 3100 mass detector, a Waters SFO system fluidics organiser, a Water 2545 binary gradient module, a Waters reagent manager and a Waters 2767 sample manager) using a SunFireTM C18 column (4.6 mm × 50 mm, 5 µm particle size): solvent gradient = 60% (or 95%) A at 0 min, 1% A at 5 min. Solvent A = 0.035% TFA in H_2_O; solvent B = 0.035% TFA in MeOH; flow rate 3.0 (or 2.5) mL/min. ^1^H and 13 C NMR spectra were obtained by using a Bruker 400 MHz FT-NMR (400 MHz for ^1^H, and 100 MHz for 13 C) spectrometer. Standard abbreviations are used for denoting the signal multiplicities.

#### 5-bromo-2-oxo-1,2-dihydropyridine-3-carbonitrile (2)

To a solution of 2-oxo-1,2-dihydropyridine-3-carbonitrile (15 g, 124.9 mmol) in acetic acid (93 mL, 0.5 M) was added Br_2_ (9.6 mL, 374.7 mmol) slowly under nitrogen atmosphere. The reaction mixture was stirred for 5 h at room temperature, diluted with Na_2_S_2_O_3_ aqueous solution and extracted with EtOAc. The organic layer was dried over Na_2_SO_4_, filtered, and concentrated to afford **2** (21.1 g, 85%) as a yellow solid. ^1^H NMR (400 MHz, DMSO-*d*_6_) δ 12.86 (bs, 1H), 8.39 (d, *J* = 2.8 Hz, 1H), 8.09 (d, *J* = 2.8 Hz, 1H). LCMS (ESI) *m/z*: 200.22 [M + H]^+^.

#### 5-((3-Fluorophenyl)thio)-2-oxo-1,2-dihydropyridine-3-carbonitrile (3)

A mixture of Compound **2** (450 mg, 2.26 mmol), 3-fluorobenzenethiol (0.25 mL, 2.34 mmol), K_2_CO_3_ (500 mg, 3.62 mmol), and copper iodide (900 mg, 4.73 mmol) in dry DMF (4.5 mL) was stirred at 120 °C for 15 h. The reaction mixture was cooled to room temperature, filtered, quenched with water, and extracted by DCM. The organic layer was dried over Na_2_SO_4_, filtered and concentrated. The resulting crude product was subjected to flash column chromatography on silica gel (10–50% EtOAc/hexane) to afford **3** (500 mg, 90%) as yellow solid. ^1^H NMR (400 MHz, DMSO-*d*_6_) δ 13.01 (bs, 1H), 8.32 (d, *J* = 2.6 Hz, 1H), 8.13 (d, *J* = 2.6 Hz, 1H), 7.39–7.33 (m, 1H), 7.10–7.01 (m, 3H). LCMS (ESI) *m/z*: 247.22 [M + H]^+^.

#### 2-Chloro-5-((3-fluorophenyl)thio)nicotinonitrile (4)

To a solution of compound **3** (620 mg, 2.52 mmol) in POCl_3_ (3.9 mL, 0.04 mmol) was added PCl_5_ (1.47 g, 7 mmol) under nitrogen atmosphere at 0 °C. The reaction mixture was stirred at 100 °C for 5 h, cooled to room temperature, diluted with water, and extracted with DCM. The organic layers were dried over Na_2_SO_4_, filtered, and concentrated. The resulting residue was subjected to silica gel column chromatography (50% EtOAc/hexane) to afford **4** (620 mg, 93%) as a brown solid. ^1^H NMR (400 MHz, DMSO-*d*_6_) δ 8.64 (d, *J* = 2.4 Hz, 1H), 8.54 (d, *J* = 2.4 Hz, 1H), 7.49–7.43 (m, 1H), 7.34–7.30 (m, 1H), 7.27 (d, *J* = 7.8 Hz, 1H), 7.24–7.19 (m, 1H). LCMS (ESI) *m/z*: 265.27 [M + H]^+^.

#### 2-Chloro-5-((3-fluorophenyl)sulfonyl)nicotinonitrile (5)

To a solution of compound **4** (600 mg, 2.27 mmol) in dry DCM (5 mL, 0.45 M) was added *m-*CPBA (1.37 g, 3.5 mmol) under nitrogen atmosphere at 0 °C. The reaction mixture was stirred at room temperature for 5 h, diluted with Na_2_SO_3_ saturated aqueous solution, and extracted with DCM. The organic layer was washed with brine, dried over Na_2_SO_4_, filtered and concentrated. The resulting residue was subjected to silica gel column chromatography (30–50% EtOAc/hexane) to afford **5** (530 mg, 79%) as a yellow solid. ^1^H NMR (400 MHz, DMSO-*d*_6_) δ 9.27 (d, *J* = 2.4 Hz, 1H), 9.17 (d, *J* = 2.4 Hz, 1H), 7.99 (dd, *J* = 8.3, 1.6 Hz, 1H), 7.94 (d, *J* = 7.8 Hz, 1H), 7.74 (td, *J* = 8.0, 5.6 Hz, 1H), 7.67–7.62 (m, 1H). LCMS (ESI) *m/z*: 297.22 [M + H]^+^.

#### 2–(5-((3-Fluorophenyl)sulfonyl)-1H-pyrazolo[3,4-b]pyridin-3-yl)isoindoline-1,3-dione (6)

A mixture of compound **5** (520 mg, 1.75 mmol), and hydrazine monohydrate (0.22 mL, 4.54 mmol) in EtOH (3 mL, 0.6 M) was stirred at 80 °C for 7 h, then cooled to room temperature, diluted with water, and extracted with DCM. The organic layer was dried over Na_2_SO_4_, filtered, and concentrated. The resulting crude product was diluted with 1,4-dioxane (7 mL) and treated with phthalic anhydride (360 mg, 2.43 mmol). The mixture was stirred at 100 °C for 15 h, filtered and concentrated. The residue was subjected to flash column chromatography on silica gel (30–70% EtOAc/hexane) to afford **6** (530 mg, 72%) as a white solid. ^1^H NMR (400 MHz, DMSO-*d*_6_) δ 14.70 (bs, 1H), 9.20 (d, *J* = 2.4 Hz, 1H), 9.10 (d, *J* = 2.0 Hz, 1H), 8.07–8.04 (m, 2H), 8.00–7.97 (m, 2H), 7.89–7.86 (m, 1H), 7.85 (d, *J* = 8.0 Hz, 1H), 7.2–7.66 (m, 1H), 7.59–7.57 (m, 1H). LCMS (ESI) *m/z*: 423.22 [M + H]^+^.

#### 2–(5-((3-Fluorophenyl)sulfonyl)-1-trityl-1H-pyrazolo[3,4-b]pyridin-3-yl)isoindoline-1,3-dione (7)

To a solution of **6** (600 mg, 1.42 mmol) and TEA (0.4 mL, 2.87 mmol) in dry DCM (22 mL) was added triphenylmethyl chloride (520 mg, 1.87 mmol) slowly at 0 °C. The mixture was stirred for 0.5 h, quenched with water, and extracted by DCM. The organic layers were dried over Na_2_SO_4_, filtered, and concentrated. The resulting residue was subjected to silica gel column chromatography (25–50% EtOAc/hexane) to afford **7** (700 mg, 75%) as a yellow solid. ^1^H NMR (400 MHz, DMSO-*d*_6_) δ 9.04 (d, *J* = 2.4 Hz, 1H), 8.95 (d, *J* = 2.4 Hz, 1H), 8.04–8.01 (m, 2H), 7.98–7.95 (m, 2H), 7.92–7.82 (m, 2H), 7.71–7.65 (m, 1H), 7.60–7.55 (m, 1H), 7.30–7.06 (m, 15H). LCMS (ESI) *m/z*: 665.27 [M + H]^+^.

#### 5-((3-Fluorophenyl)sulfonyl)-1-trityl-1H-pyrazolo[3,4-b]pyridin-3-amine (8)

A suspension of compound **7** (1 g, 1.5 mmol) and hydrazine monohydrate (0.22 mL, 4.5 mmol) in THF (15 mL) and EtOH (15 mL) was stirred at room temperature for 1 h. The mixture was concentrated and the generated residue was subjected to silica gel column chromatography (20–50% EtOAc/hexane) to afford **8** (680 mg, 85%) as a pale yellow solid. ^1^H NMR (400 MHz, DMSO-*d*_6_) δ 8.23 (d, *J* = 2.4 Hz, 1H), 8.68 (d, *J* = 2 Hz, 1H), 7.84–7.79 (m, 2H), 7.71–7.65 (m, 1H), 7.58–7.54 (m, 1H), 7.31–7.14 (m, 15H), 6.27 (bs, 2H). 13 C NMR (100 MHz, DMSO-*d*_6_) δ 163.59, 161.10, 153.83, 149.59, 146.87, 143.96, 143.89, 143.16, 132.77, 132.69, 131.61, 129.47, 128.66, 127.90, 127.74, 126.95, 123.89, 123.86, 121.52, 121.31, 120.97, 114.7, 114.63, 109.67, 77.55. LCMS (ESI) *m/z*: 535.27 [M + H]^+^.

#### 4–(1,3-Dioxoisoindolin-2-yl)-N-(5-((3-fluorophenyl)sulfonyl)-1-trityl-1H-pyrazolo[3,4-b]pyridin-3-yl)benzamide (9i)

To a solution of **8** (500 mg, 0.94 mmol) and DMAP (50 mg, 0.41 mmol) in pyridine (10 mL) was added 4–(1,3-dioxoisoindolin-2-yl)benzoyl chloride (400 mg, 1.4 mmol) dropwise at 0 °C under nitrogen atmosphere. The mixture was stirred at room temperature for 1 h, then diluted with water, and extracted with DCM. The organic layer was dried over Na_2_SO_4_, filtered and concentrated. The resulting crude was subjected to silica gel column chromatography (25–80% EtOAc/hexane) to afford **9i** (680 mg, 92%) as a yellow solid. ^1^H NMR (400 MHz, DMSO-*d*_6_) δ 11.53 (bs, 1H), 9.03 (s, 1H), 8.85 (s, 1H), 8.21 (d, *J* = 8.4 Hz, 2H), 8.02–8.00 (m, 2H), 7.95–7.93 (m, 2H), 7.88 (d, *J* = 8.0 Hz, 1H), 7.71–7.63 (m, 3H), 7.59–7.55 (m, 1H), 7.31–7.21 (m, 15H). LCMS (ESI) *m/z*: 784.27 [M + H]^+^.

#### 4-Amino-N-(5-((3-fluorophenyl)sulfonyl)-1-trityl-1H-pyrazolo[3,4-b]pyridin-3-yl)benzamide (11)

A mixture of compound **9i** (200 mg, 0.26 mmol) and hydrazine monohydrate (0.06 mL, 1.3 mmol) in THF (4 mL) and EtOH (4 mL) was stirred at room temperature for 1 h. The mixture was concentrated and the generated residue was subjected to silica gel column chromatography (50–80% EtOAc/hexane) to afford **11** (150 mg, 88%) as a pale yellow solid. ^1^H NMR (400 MHz, DMSO-*d*_6_) δ 10.86 (bs, 1H), 8.93 (d, *J* = 2.4 Hz, 1H), 8.81 (d, *J* = 2.4 Hz, 1H), 7.93–7.90 (m, 1H), 7.87–7.82 (m, 3H), 7.69–7.64 (m, 1H), 7.58–7.53 (m, 1H), 7.27–7.18 (m, 15H), 6.59 (d, *J* = 8.8 Hz, 2H), 5.87 (bs, 2H). 13 C NMR (100 MHz, DMSO-*d*_6_) δ 165.87, 163.47, 160.99, 153.10, 152.46, 146.95, 143.37, 143.30, 142.57, 141.92, 134.88, 132.62, 132.55, 130.53, 129.68, 129.54, 127.85, 127.23, 124.10, 124.07, 121.62, 121.41, 119.24, 115.11, 114.87, 112.77, 110.85, 78.36. LCMS (ESI) *m/z*: 654.22 [M + H]^+^.

#### General procedure A for the synthesis of compounds 10a–10h

To a solution of **8** (1 equiv) in pyridine (0.1 M) was added various acid chlorides (1.05 equiv) and DMAP (0.6 equiv) at 0 °C under nitrogen atmosphere. The mixture was stirred at room temperature for 0.5 h, then diluted with water, and extracted with DCM. The organic layer was dried over Na_2_SO_4_, filtered, and concentrated. The residue was diluted with dry DCM (0.5 M), and added slowly with TFA (2 equiv) at 0 °C. The mixture was stirred at room temperature for 0.5 h, diluted with water, and extracted with DCM. The organic phase was washed with brine, dried over Na_2_SO_4_, filtered and concentrated. The resulting crude product was subjected to flash column chromatography on silica gel.

#### 4-Cyano-N-(5-((3-fluorophenyl)sulfonyl)-1H-pyrazolo[3,4-b]pyridin-3-yl)benzamide (10a)

General procedure A was used to transform **8** (50 mg, 0.09 mmol) to the target compound. The resulting residue was subjected to flash column chromatography on silica gel (50–100% THF/hexane) to afford **10a** (14.2 mg, 37%) as a yellow solid. ^1^H NMR (400 MHz, DMSO-*d*_6_) δ 14.09 (bs, 1H), 11.67 (bs, 1H), 9.15 (s, 1H), 9.10 (s, 1H), 8.24 (d, *J* = 8.0 Hz, 2H), 8.06 (d, *J* = 8.0 Hz, 2H), 7.93 (d, *J* = 8.4 Hz, 1H), 7.89 (d, *J* = 7.6 Hz, 1H), 7.72–7.66 (m, 1H), 7.59–7.55 (m, 1H). 13 C NMR (100 MHz, DMSO-*d*_6_) δ 164.79, 163.67, 161.19, 152.70, 148.62, 143.96, 143.89, 141.84, 137.79, 135.33, 132.96, 132.82, 132.74, 129.51, 129.44, 124.03, 124.00, 121.62, 121.41, 118.72, 115.00, 114.87, 114.76, 107.71. LCMS (ESI) *m/z*: 422.27 [M + H]^+^.

#### N-(5-((3-Fluorophenyl)sulfonyl)-1H-pyrazolo[3,4-b]pyridin-3-yl)-3-(trifluoromethyl)benzamide (10b)

General procedure A was used to transform **8** (50 mg, 0.09 mmol) to the target compound. The resulting residue was subjected to flash column chromatography on silica gel (40–90% THF/hexane) to afford **10 b** (21.5 mg, 51%) as a white solid. ^1^H NMR (400 MHz, DMSO-*d*_6_) δ 14.08 (bs, 1H), 11.69 (bs, 1H), 9.16 (d, *J* = 2.0 Hz, 1H), 9.10 (d, *J* = 2.4 Hz, 1H), 8.46 (bs, 1H), 8.39 (d, *J* = 7.6 Hz, 1H), 8.02 (d, *J* = 8 Hz, 1H), 7.94–7.91 (m, 1H), 7.89 (d, *J* = 8.0 Hz, 1H), 7.82 (t, *J* = 7.6 Hz, 1H), 7.72–7.66 (m, 1H), 7.59–7.54 (m, 1H). 13 C NMR (100 MHz, DMSO-*d*_6_) δ 165.11, 163.51, 161.02, 152.54, 148.37, 143.83, 143.76, 142.47, 142.20, 135.28, 132.65, 132.57, 130.35, 130.08, 129.08, 127.81, 123.85, 121.43, 121.22, 117.99, 114.82, 114.58, 112.87, 107.60. LCMS (ESI) *m/z*: 465.22 [M + H]^+^.

#### N-(5-((3-Fluorophenyl)sulfonyl)-1H-pyrazolo[3,4-b]pyridin-3-yl)-4-(trifluoromethyl)benzamide (10c)

General procedure A was used to transform **8** (50 mg, 0.09 mmol) to the target compound. The resulting residue was subjected to flash column chromatography on silica gel (40–90% THF/hexane) to afford **10c** (18.7 mg, 45%) as a white solid. ^1^H NMR (400 MHz, DMSO-*d*_6_) δ 14.07 (bs, 1H), 11.65 (bs, 1H), 9.16 (d, *J* = 2.0 Hz, 1H), 9.10 (d, *J* = 1.6 Hz, 1H), 8.29 (d, *J* = 8.0 Hz, 2H), 7.94 (d, *J* = 8.4 Hz, 2H), 7.92–7.88 (m, 2H), 7.72–7.66 (m, 1H), 7.59–7.56 (m, 1H). 13 C NMR (100 MHz, DMSO-*d*_6_) δ 164.92, 163.61, 161.13, 152.65, 148.55, 143.91, 143.85, 141.86, 137.50, 135.27, 132.75, 132.67, 132.43, 132.11, 129.56, 129.36, 125.85, 125.81, 125.78, 125.64, 123.96, 123.93, 122.93, 121.55, 121.34, 114.93, 114.69, 107.65. LCMS (ESI) *m/z*: 465.22 [M + H]^+^.

#### N-(5-((3-Fluorophenyl)sulfonyl)-1H-pyrazolo[3,4-b]pyridin-3-yl)-4-methoxybenzamide (10d)

General procedure A was used to transform **8** (50 mg, 0.09 mmol) to the target compound. The resulting residue was subjected to flash column chromatography on silica gel (30–70% THF/hexane) to afford **10d** (13.3 mg, 35%) as a white solid.1H NMR (400 MHz, DMSO-*d*_6_) δ 13.96 (bs, 1H), 11.24 (bs, 1H), 9.10 (d, *J* = 17.6 Hz, 2H), 8.11 (d, *J* = 7.6 Hz, 2H), 7.93–7.88 (m, 2H), 7.69–7.68 (m, 2H), 7.58–7.56 (m, 1H), 7.09 (d, *J* = 8 Hz, 2H), 3.86 (s, 3H). 13 C NMR (100 MHz, DMSO-*d*_6_) δ 165.24, 163.52, 162.73, 152.57, 148.35, 143.86, 143.80, 135.32, 132.66, 132.58, 130.56, 129.05, 125.47, 123.84, 121.44, 121.22, 114.84, 114.59, 114.04, 107.65, 55.81. LCMS (ESI) *m/z*: 426.27 [M + H]^+^.

#### 4-(Dimethylamino)-N-(5-((3-fluorophenyl)sulfonyl)-1H-pyrazolo[3,4-b]pyridin-3-yl)benzamide (10e)

General procedure A was used to transform **8** (50 mg, 0.09 mmol) to the target compound. The resulting residue was subjected to flash column chromatography on silica gel (50–90% THF/hexane) to afford **10e** (10.1 mg, 26%) as a yellow solid. ^1^H NMR (400 MHz, DMSO-*d*_6_) δ 13.91 (bs, 1H), 10.99 (bs, 1H), 9.12 (d, *J* = 2.0 Hz, 1H), 9.06 (d, *J* = 2.0 Hz, 1H), 8.01 (d, *J* = 8.8 Hz, 2H), 7.92 (d, *J* = 8.4 Hz, 1H), 7.89 (d, *J* = 8.0 Hz, 1H), 7.71–7.65 (m, 1H), 7.58–7.53 (m, 1H), 6.77 (d, *J* = 8.8 Hz, 2H), 3.02 (s, 6H). 13 C NMR (100 MHz, DMSO-*d*_6_) δ 165.64, 163.69, 161.21, 153.26, 152.75, 148.45, 144.08, 144.02, 142.91, 135.69, 132.82, 132.75, 130.28, 129.06, 124.00, 121.58, 121.37, 119.55, 114.98, 114.74, 111.25, 107.85, 67.50, 49.08, 34.86, 30.64, 25.60. LCMS (ESI) *m/z*: 440.27 [M + H]^+^.

#### N-(5-((3-Fluorophenyl)sulfonyl)-1H-pyrazolo[3,4-b]pyridin-3-yl)-4-morpholinobenzamide (10f)

General procedure A was used to transform **8** (50 mg, 0.09 mmol) to the target compound. The resulting residue was subjected to flash column chromatography on silica gel (50–90% THF/hexane) to afford **10f** (11.5 mg, 27%) as a white solid. ^1^H NMR (400 MHz, DMSO-*d*_6_) δ 13.95 (bs, 1H), 11.10 (bs, 1H), 9.12 (s, 1H), 9.07 (s, 1H), 8.03 (d, *J* = 8.0 Hz, 2H), 7.93–7.88 (m, 2H), 7.69–7.68 (m, 1H), 7.58–7.56 (m, 1H), 7.04 (d, *J* = 8.0 Hz, 2H), 3.75 (bs, 4H), 3.29 (m, 4H). 13 C NMR (100 MHz, DMSO-*d*_6_) δ 165.44, 16.68, 161.20, 154.03, 152.73, 148.47, 144.05, 143.99, 142.70, 135.59, 132.80, 132.72, 130.18, 129.11, 123.98, 122.54, 121.57, 121.36, 114.98, 114.74, 113.69, 107.81, 66.37, 47.57. LCMS (ESI) *m/z*: 482.27 [M + H]^+^.

#### N-(5-((3-Fluorophenyl)sulfonyl)-1H-pyrazolo[3,4-b]pyridin-3-yl)-4–(4-methylpiperazin-1-yl)benzamide (10g)

General procedure A was used to transform **8** (50 mg, 0.09 mmol) to the target compound. The resulting residue was subjected to flash column chromatography on silica gel (50–100% THF/hexane) to afford **10 g** (16 mg, 36%) as a white solid. ^1^H NMR (400 MHz, DMSO-*d*_6_) δ 13.94 (bs, 1H), 11.07 (bs, 1H), 9.12 (d, *J* = 2.4 Hz, 1H), 9.07 (d, *J* = 2.4 Hz, 1H), 8.00 (d, *J* = 8.8 Hz, 2H), 7.93–7.88 (m, 2H), 7.71–7.66 (m, 1H), 7.58–7.53 (m, 1H), 7.03 (d, *J* = 8.8 Hz, 2H), 3.33–3.31 (m, 4H), 2.46–2.43 (m, 4H), 2.23 (s, 3H). 13 C NMR (100 MHz, DMSO-*d*_6_) δ 164.94, 163.16, 160.68, 153.35, 152.21, 147.94, 143.54, 143.48, 142.21, 135.08, 132.28, 132.21, 129.69, 128.58, 123.49, 121.48, 121.05, 120.83, 114.46, 114.21, 113.29, 107.30, 79.12, 64.85, 54.29, 46.70, 45.68, 15.11. LCMS (ESI) *m/z*: 495.27 [M + H]^+^.

#### N-(5-((3-Fluorophenyl)sulfonyl)-1H-pyrazolo[3,4-b]pyridin-3-yl)-3–(4-methylpiperazin-1-yl)benzamide (10h)

General procedure A was used to transform **8** (50 mg, 0.09 mmol) to the target compound. The resulting residue was subjected to flash column chromatography on silica gel (50–100% THF/hexane) to afford **10 h** (9 mg, 20%) as a white solid. ^1^H NMR (400 MHz, DMSO-*d*_6_) δ 11.35 (bs, 1H), 9.13 (d, *J* = 2.0 Hz, 1H), 9.08 (d, *J* = 2.4 Hz, 1H), 7.95–7.91 (m, 1H), 7.89 (d, *J* = 8.0 Hz, 1H), 7.71–7.66 (m, 1H), 7.64 (bs, 1H), 7.59–7.54 (m, 1H), 7.5 (d, *J* = 7.6 Hz, 1H), 7.38 (t, *J* = 8.4 Hz, 1H), 7.21–7.18 (m, 1H), 3.26–3.24 (m, 4H), 2.48–2.47 (m, 4H), 2.24 (s, 3H). LCMS (ESI) *m/z*: 495.27 [M + H]^+^.

#### 4-Amino-N-(5-((3-fluorophenyl)sulfonyl)-1H-pyrazolo[3,4-b]pyridin-3-yl)benzamide (12)

To a solution of **11** (50 mg, 0.076 mmol) in DCM (1 mL) was added TFA (0.1 mL, 0.15 mmol) dropwise. The mixture was stirred at room temperature for 0.5 h, diluted with water, and extracted with DCM. The organic phase was washed with brine, dried over Na_2_SO_4_, filtered and concentrated. The resulting crude product was subjected to flash column chromatography on silica gel (40–80% THF/hexane) to afford **12** (16 mg, 51%) as a pale yellow solid. ^1^H NMR (400 MHz, DMSO-*d*_6_) δ 13.89 (bs, 1H), 10.87 (bs, 1H), 9.06 (d, *J* = 6.0 Hz, 2H), 7.93–7.84 (m, 4H), 7.71–7.65 (m, 1H), 7.58–7.54 (m, 1H), 6.61 (d, *J* = 8.4 Hz, 2H), 5.87 (bs, 2H). 13 C NMR (100 MHz, DMSO-*d*_6_) δ 165.70, 163.68, 161.19, 153.22, 152.73, 148.41, 144.09, 144.02, 142.94, 135.63, 132.80, 132.72, 130.52, 129.00, 124.00, 123.97, 121.55, 121.34, 119.58, 114.96, 114.72, 113.04, 107.85, 31.15. LCMS (ESI) *m/z*: 412.27 [M + H]^+^.

#### General procedure B for the synthesis of compounds 13a–13c

To a solution of **11** (1 equiv) in pyridine (0.1 M) was added various acid chlorides (1.05 equiv) and DMAP (0.6 equiv) at 0 °C under nitrogen atmosphere. The mixture was stirred at room temperature for 0.5 h, then diluted with water, and extracted with DCM. The organic layer was dried over Na_2_SO_4_, filtered, and concentrated. The residue was diluted with dry DCM (0.5 M) and treated slowly with TFA (2 equiv) at 0 °C. The mixture was stirred at room temperature for 0.5 h, diluted with water, and extracted with DCM. The organic phase was washed with brine, dried over Na_2_SO_4_, filtered and concentrated. The resulting crude product was subjected to flash column chromatography on silica gel.

#### 4–(3,3-Dimethylureido)-N-(5-((3-fluorophenyl)sulfonyl)-1H-pyrazolo[3,4-b]pyridin-3-yl)benzamide (13a)

General procedure B was used to transform **11** (50 mg, 0.076 mmol) to the target compound. The resulting residue was subjected to flash column chromatography on silica gel (50–100% THF/hexane) to afford **13a** (16 mg, 43%) as a white solid. ^1^H NMR (400 MHz, DMSO-*d*_6_) δ 13.96 (bs, 1H), 11.18 (bs, 1H), 9.13 (d, *J* = 2.4 Hz, 1H), 9.08 (d, *J* = 2.0 Hz, 1H), 8.64 (bs, 1H), 8.02 (d, *J* = 8.8 Hz, 2H), 7.94–7.88 (m, 2H), 7.71–7.65 (m, 3H), 7.59–7.56 (m, 1H), 2.96 (bs, 6H). 13 C NMR (100 MHz, DMSO-*d*_6_) δ 165.54, 163.67, 161.19, 155.78, 152.72, 148.49, 145.12, 144.04, 143.97, 142.54, 135.51, 132.81, 132.73, 129.34, 129.18, 125.94, 124.01, 123.99, 121.58, 121.37, 118.70, 114.98, 114.73, 107.80, 36.75. LCMS (ESI) *m/z*: 483.27 [M + H]^+^.

#### N-(5-((3-Fluorophenyl)sulfonyl)-1H-pyrazolo[3,4-b]pyridin-3-yl)-4-propionamidobenzamide (13b)

General procedure B was used to transform **11** (50 mg, 0.076 mmol) to the target compound. The resulting residue was subjected to flash column chromatography on silica gel (50–100% THF/hexane) to afford **13b** (18 mg, 50%) as a white solid. ^1^H NMR (400 MHz, DMSO-*d*_6_) δ 13.99 (bs, 1H), 11.26 (bs, 1H), 10.19 (bs, 1H), 9.13 (d, *J* = 2.4 Hz, 1H), 9.08 (d, *J* = 2.0 Hz, 1H), 8.08 (d, *J* = 8.8 Hz, 2H), 7.94–7.91 (m, 1H), 7.89 (d, *J* = 8 Hz, 1H), 7.76 (d, *J* = 8.4 Hz, 2H), 7.71–7.66 (m, 1H), 7.58–7.56 (m, 1H), 2.38 (q, *J* = 7.6 Hz, 2H), 1.10 (tr, *J* = 7.6 Hz, 3H). 13 C NMR (100 MHz, DMSO-*d*_6_) δ 172.91, 165.30, 163.57, 161.09, 152.61, 148.41, 143.92, 143.85, 143.28, 142.34, 135.38, 132.69, 132.61, 129.60, 129.12, 127.42, 123.91, 123.88, 121.47, 121.26, 118.49, 114.88, 114.64, 107.69, 30.00, 9.85. LCMS (ESI) *m/z*: 468.27 [M + H]^+^.

#### N-(5-((3-Fluorophenyl)sulfonyl)-1H-pyrazolo[3,4-b]pyridin-3-yl)-4-(methylsulfonamido)benzamide (13c)

General procedure B was used to transform **11** (50 mg, 0.076 mmol) to the target compound. The resulting residue was subjected to flash column chromatography on silica gel (50–100% THF/hexane) to afford **13c** (12 mg, 32%) as a pale yellow solid. ^1^H NMR (400 MHz, DMSO-*d*_6_) δ 14.01 (bs, 1H), 11.30 (bs, 1H), 9.12 (d, *J* = 2.0 Hz, 1H), 9.08 (d, *J* = 2.0 Hz, 1H), 8.10 (d, *J* = 8.4 Hz, 2H), 7.93–7.88 (m, 2H), 7.71–7.66 (m, 1H), 7.59–7.54 (m, 1H), 7.32 (d, J = 8.4 Hz, 2H), 3.12 (s, 3H). 13 C NMR (100 MHz, DMSO-*d*_6_) δ 164.69, 161.19, 152.72, 148.61, 143.98, 143.91, 141.98, 135.40, 134.63, 132.82, 132.74, 130.28, 129.87, 129.55, 129.40, 129.21, 125.42, 125.37, 124.02, 121.61, 121.41, 114.99, 114.74, 107.74. LCMS (ESI) *m/z*: 490.27 [M + H]^+^.

### Cell culture and reagent

ALK wt-TEL, ALK L1196M-TEL transformed Ba/F3 and H2228 cell lines were cultured in RPMI1640 (Welgene, # LM011-01). The culture media was supplemented with 10% foetal bovine serum (Hyclone), Antibiotic-Antimycotic solution (Welgene, # LS203-01) containing 10,000 U/mL penicillin, 10 mg/mL streptomycin and 25 µg/mL amphotericin B in 0.85% NaCl. Parental Ba/F3 cells were cultured in RPMI1640 media supplemented with 10% foetal bovine serum and 1% penicillin/streptomycin solution in the presence of IL-3. The cells were maintained in a humidified atmosphere containing 5% CO_2_ at 37 °C.

### Biochemical kinase assay

Biochemical kinase assay was performed by methods previously reported^39^. Kinase reactions of all test compounds except **10g** were carried out at 10 µM ATP. Kinase reaction of **10g** was performed at three different concentrations of ATP (10/50/100 µM).

### Anti-proliferation assay

Cells (ALK wt-TEL, ALK L1196M-TEL and Parental Ba/F3: 1 × 10^4^, H2228: 5 × 10^3^) were plated in 96 well tissue culture plates. Each compound was added to each well at 10 dose points of three-fold serial dilution in DMSO. After treatment with each compound for 72 h, CTG assay solution (Promega, # G7572) was added to each well. Cell proliferation was assessed by measuring the luminescence using a 96 well plate reader (EnVision 2013).

### Western blot

Cells were harvested and lysed using IP buffer containing 50 mM HEPES (pH 7.4), 1% Triton X-100, 2 mM EDTA, 150 mM NaCl, 2.5 mM NaF, 5 mM Na_3_VO_4_, protease inhibitor cocktail tablet (Roche, # 11–878-580–001). The protein concentration was determined by Bradford assay. Proteins were separated using SDS-PAGE and transferred onto NC membrane. The membranes were blocked using 5% skim milk in TBS-T buffer. The rabbit polyclonal antibody against phospho-ALK (Tyr1604, # 3341), phospho-ERK1/2 (Thr202/Tyr204, # 8544), phospho- PLC-gamma (Tyr783, # 14008), phospho-STAT3 (Tyr705, # 4074) and cleaved caspase3 (# 9661) were purchased from Cell Signalling Technologies, and PARP1/2 (H-250, # sc-7150) and anti-β-actin (# sc-47778) antibody was obtained from Santa Cruz Biotechnology. All primary antibodies were diluted in TBS-T at 1:1000. Each primary antibody was incubated overnight at 4 °C, followed by the secondary antibody treatment for 1 h at room temperature. Secondary antibodies were purchased from genDEPOT. Proteins were detected using ECL substrate, and then exposed to X-ray film.

### Apoptosis analysis and cell cycle arrest

After 24–48 h following compound treatment, cells were harvested and stained with annexin V (1:500 diluted in annexin V buffer) for 30 min and PI (1:200) solution for 30 min. 1 × 10^6^ cells were analysed using flow cytometric analysis. For the cell cycle arrest analysis, cells were harvested by trypsinisation and fixed with 70% ethanol overnight at −20 °C. The next day, cells were harvested and washed with cold DPBS at 500 × g. The cells were suspended in RNase/PI solution (Cell Signalling Technologies, # 4087).

### Permeability assessment for 10g using Caco-2 cells

Stock solutions (10 mM) of reference compounds and **10g** were diluted to a concentration of 10 µM with the transport buffer (HBSS + 1%BSA) and the test compounds were applied to the apical or basolateral side of the cell monolayer. Permeability of the test compounds from A to B direction or B to A direction was assessed in duplicate over a 120 min incubation at 37 °C and 5% CO_2_ with a relative humidity of 95%. In addition, the efflux ratio of each compound was also determined. Reference compounds and **10g** were quantified by LC-MS/MS analysis based on the peak area ratio of analyte/the internal standard.

### Molecular docking study

X-ray co-crystal structures of ALK kinase domain complexed with crizotinib (PDB code: 2XP2) and ALK kinase domain in complex with entrectinib (PDB code: 5FTO) were retrieved from Protein Data Bank. Molecular docking studies were carried out by methods previously reported^40^.

### Statistics

Statistical analysis was performed using GraphPad Prism (Ver 6.01). All values are expressed as the mean standard deviation.

## Results and discussion

### Chemistry

As shown in Scheme 1, synthesis of the pyrazolo[3,4-b]pyridine derivatives commenced with bromination of commercially available 2-oxo-1,2-dihydropyridine-3-carbonitrile **1** using bromine in AcOH to give bromide **2**, which was substituted with 3-fluorobenzenethiol using CuI and K_2_CO_3_ to afford **3** in 90% yield. Sulphide **3** was treated with PCl_5_/POCl_3_ to give the chloride **4** (93%), which was oxidised using *m-*CPBA to furnish the sulfone **5**. Subjection of **5** to aminopyrazole ring formation conditions using hydrazine followed by protection of the resulting amine group with phthalic anhydride yielded **6** in 72% yield. The pyrazole moiety of **6** was protected with a trityl group to give **7** and phthalimide protecting group of **7** was removed using hydrazine to afford the aminopyrazole **8**. Amide coupling reactions of **8** with various benzoyl chlorides were performed in the presence of DMAP to produce the amides **9a-i**. The trityl groups in **9a-h** and **11** were removed using TFA to yield the target the pyrazolo[3,4-b]pyridine derivatives **10a-h** and **12**, respectively. The aniline **11** was transformed to the corresponding urea, amide and sulfonamide followed by removal of trityl group to give the target derivatives **13a-c**.

**Figure:**
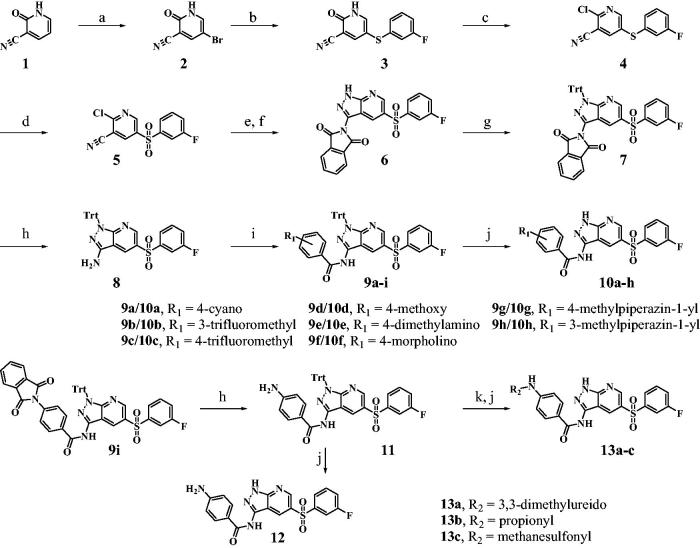
**Scheme 1**. Synthesis of derivatives **10a-h**, **12**, **13a-c**^a^. ^a^Reagents and conditions: (a) Br_2_, Acetic acid, rt (85%); (b) 3-fluorobenzenethiol, copper iodide, K_2_CO_3_, DMF, 120 °C (90%); (c) PCl_5_, POCl_3_, 100 °C (93%); (d) *m-*CPBA, Na_2_SO_3_, DCM, 0 °C (79%); (e) hydrazine monohydrate, EtOH, 80 °C; (f) phthalic anhydride, 1,4-dioxane, 100 °C (72% over 2 steps); (g) triphenylmethyl chloride, TEA, DCM_,_ 0 °C (75%); (h) hydrazine monohydrate, THF, EtOH, rt (85%); (i) various benzoyl chloride, DMAP, pyridine, 0 °C (70–85%); (j) TFA, DCM, rt (35–45% over 2 steps); (k) for **13a**, dimethylcarbamic chloride, DMAP, pyridine, 0 °C (43%); for **13b**, propionyl chloride, DMAP, pyridine, 0 °C (50%); for **13c**, methanesulfonyl chloride, DMAP, pyridine, 0 °C (32%).

### Rationale for the design of pyrazolo[3,4-b]pyridines and SAR study

The goal of the investigation described below was to identify unique and potent inhibitors against both ALK-wt and ALK-L1196M. Based on the observation that entrectinib^11^ strongly inhibits ALK-L1196M^41^, we selected the indazole platform design strategy because substances in this family have not been widely explored in efforts focusing on the discovery of ALK inhibitors while they have been investigated^11^ in our other kinase programs. Additional considerations led us to choose the closely related pyrazolo[3,4-b]pyridine group, which is superior to indazole in terms of cLogP, as the core structure. It should be noted that ALK inhibition properties of this type of substances have not been elucidated previously. It was reported^42^ that a fluorine group at the C3 position of the phenyl group in crizotinib contributes to potency and binding efficiency, and that entrectinib contains a related fluorophenyl moiety. These findings prompted us to incorporate a 3-fluorophenyl group in the newly designed pyrazolo[3,4-b]pyridine based ALK inhibitors. Moreover, we envisioned that the methylene linker between indazole core and difluorophenyl ring of entrectinib could be optimised to promote additional interaction with ALK. In addition, the results of docking studies suggested that the methylene linker in entrectinib could be replaced by a sulfone group. These considerations led us to design a new family potential ALK inhibitors that contain a common 5-((3-fluorophenyl)sulfonyl)-1H-pyrazolo[3,4-b]pyridine moiety and various head groups that should reside in solvent exposed region.

The inhibitory activities of the pyrazolopyridine derivatives against both ALK-wt and ALK-L1196M were assessed by using an *in vitro* biochemical assay. As shown in Table 1, the kinase-inhibitory activities of the derivatives were highly dependent on the R_1_ group. For example, **10a** containing a 4-cyano group displayed a reasonable inhibitory activity (IC_50_ = 453 nM) on ALK-wt. Introduction of a trifluoromethyl group as R_1_ (**10b** and **10c**) resulted in little to no activity against ALK-wt. In contrast, the 4-methoxy containing derivative **10d** has an enhanced activity against ALK-wt (IC_50_ = 69 nM) and it possesses a high activity (IC_50_ = 19 nM) against ALK-L1196M gatekeeper mutation, a value that is 50-fold higher than that (IC_50_ = 980 nM) of crizotinib. Moreover, replacement of the 4-methoxy group by a 4-dimethylamino group led to **10e**, which was found to exhibit picomolar activity against ALK-L1196M. It is worthwhile to note that **10e** is more potent against ALK-L1196M (IC_50_ = 0.7 nM) than against ALK-wt (IC_50_ = 7.3 nM). Picomolar inhibitory activity against ALK-wt was achieved with the 4-morpholino derivative **10f**, which is also extremely active against ALK-L1196M (IC_50_ = 1.4 nM). The SAR study led us to identify **10g** containing a 4-methylpiperazin-1-yl group as the most potent inhibitor against both ALK-wt (IC_50_ < 0.5 nM) and ALK-1196M (IC_50_ < 0.5 nM).

**Table 1.** Kinase-inhibitory activities of 1*H*-pyrazolo[3,4-b]pyridine derivatives against ALK-wt and ALK-L1196M.

^a^Radiometric kinase assay.

^b^‘Inactive’ means that kinase activity is inhibited by less than 50% even at 10 μM concentration of compound.

^c^Activity value from the reference^15^.

### Antiproliferative activities of selected pyrazolo[3,4-b]pyridines

Based on the results arising from studies of the kinase-inhibitory activities of the pyrazolo[3,4-b]pyridine derivatives against ALK-wt and ALK-L1196M gatekeeper mutant, we selected the most potent inhibitors and measured their antiproliferative activities on Ba/F3 cells transformed with ALK-wt/ALK-L1196M and on H2228 non-small cell lung cancer cells harbouring EML4-ALK. Ba/F3 cell lines transformed with ALK-wt and ALK-L1196M mutant were employed to assess the ALK inhibition capability of the derivatives in a cellular context and parental Ba/F3 cells were utilised as controls to determine differential cytotoxicities. The antiproliferative activities of the selected pyrazolo[3,4-b]pyridines were further elucidated using the H2228 NSCLC cell line, which is an EML4-ALK positive cancer cell line. As the data in Table 2 show, the overall cellular activities of the selected pyrazolo[3,4-b]pyridines are relatively moderate compared with their enzymatic activities. In particular, it is difficult to understand why **10d** is potent against ALK enzyme but inactive on H2228 and ALK-driven Ba/F3 cells. Among the compounds tested, **10g** most strongly suppressed proliferation of both H2228 (GI_50_ = 0.219 µM) and ALK-driven Ba/F3 cells (GI_50_ < 0.205 µM).

**Table 2.** Antiproliferative activities of 1*H*-pyrazolo[3,4-b]pyridine derivatives against Ba/F3 transformed with ALK and H2228 NSCLC cancer cell.

Entry	GI_50_ (μM)^a,b^			
	H2228 (EML4-ALK)	Ba/F3 cell lines		
		Parental	ALK wt-TEL	ALKL1196M-TEL
**crizotinib**	0.249 ± 0.06	1.654 ± 0.13	0.141 ± 0.08	0.726 ± 0.21
**10d**	Inactive	Inactive	Inactive	Inactive
**10e**	8.538 ± 0.78	Inactive	1.767 ± 0.69	4.549 ± 0.72
**10f**	1.693 ± 0.40	Inactive	0.916 ± 0.50	2.527 ± 1.50
**10g**	0.219 ± 0.05	3.495 ± 1.13	0.205 ± 0.06	0.129 ± 0.02
**10h**	Inactive	15.18 ± 0.52	3.352 ± 0.24	2.276 ± 0.59
**12**	4.033 ± 1.81	Inactive	3.869 ± 1.54	1.980 ± 0.28
**13c**	9.215 ± 1.92	inactive	4.708 ± 3.02	5.625 ± 2.34

^a^GI_50_ represents the concentration at which a compound causes half-maximal growth inhibition. GI_50_ value for parental, Ba/F3 transformed with ALK and H2228 cell lines were shown as the means ± standard deviation (SD) of three independent experiments.

^b^‘Inactive’ means that the proliferation was suppressed by less than 10% even at 50 μM concentration of compound.

In order to understand the discrepancy between enzymatic and cellular activities, we first assessed the cell permeability of **10g** using the human colon carcinoma cell line Caco-2. It was found that **10g** has moderate permeability and is not a substrate of P-glycoprotein (P-gp) as evidenced by the fact that the efflux ratio of **10g** is 1.85 (Table 3). This finding indicates that the cell permeability of **10g** is not the reason for the discrepancy. We next measured the kinase-inhibitory activities of **10g** against ALK-wt at three different ATP concentrations because the IC_50_ value derived from biochemical kinase assay depends on both *K*_i_ and *K*_m_, which are defined by ATP concentration^43^^,^^44^. As described in Table 4, it was observed that a 10-fold increase in ATP concentration resulted in a 50-fold decrease in IC_50_ (IC_50_ = 24 nM at 100 µM ATP). It should be noted that the physiological ATP concentration is around 1 mM and the IC_50_ value of **10g** should be much higher than 24 nM at 1 mM ATP concentration, which may explain the discrepancy.

**Table 3.** Cell permeability assessment of **10g** using Caco-2 cells.

	Mean Papp^a^ (10^–6^ cm/s)		Efflux ratio	Mean recovery %		Rank	
	A to B	B to A		A to B	B to A	Papp	P-gp
Atenolol	1.69	2.14	1.27	92.60	91.61	Low	
Propranolol	16.05	8.42	0.52	84.81	91.66	High	
Digoxin	0.43	8.60	20.22	96.82	95.09	Low	Substrate
**10g**	2.56	4.74	1.85	74.17	92.30	Moderate	

^a^Papp (A to B) < 2.5: Low permeability, 2.5< Papp (A to B) < 10: Moderate permeability, Papp (A to B) > 10: High permeability.

**Table 4.** IC_50_ values of **10g** at various ATP concentrations.

	IC_50_ (nM)^a^		
	ALK-wt		
	[ATP](10 μM)	[ATP](50 μM)	[ATP](100 μM)
**10g**	<0.5	17.3	24.9

The IC_50_ values of **10g** against ALK-wt were measured depending on ATP concentrations ranging from 10 to 100 μM.

^a^Radiometric biochemical kinase assay.

Furthermore, it should be emphasised that **10g** more strongly inhibits (GI_50_ = 0.129 µM) proliferation of Ba/F3 cells transformed with ALK-L1196M than crizotinib (GI_50_ = 0.726 µM). In addition, **10g** is 27-fold more potent against ALK-L1196M Ba/F3 cells than parental Ba/F3 cells while crizotinib is just 2-fold more potent against ALK-L1196M Ba/F3 cells than parental Ba/F3 cells. These findings indicate that **10g** possesses much more favourable differential cytotoxicity than does crizotinib. Pyrazolo[3,4-b]pyridine **10f**, the second most potent inhibitor of the ALK enzymes, displays high antiproliferative activities on H2228 and ALK-driven Ba/F3 cells. Like **10g**, with exception of **10d**, all of the other pyrazolo[3,4-b]pyridines tested including **10e**, **10f**, **10h**, **12**, and **13c**, exhibit higher differential cytotoxicities against ALK-L1196M Ba/F3 cells vs. parental Ba/F3 cells than does crizotinib. Based on the results of the antiproliferative activity studies, **10g** was selected for further cellular investigations.

### Kinase profile of 10g

**Figure 2.**
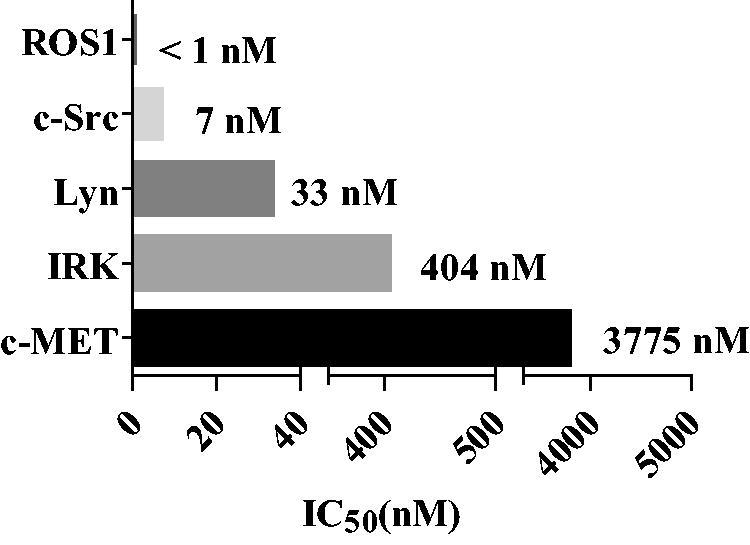
Kinase-inhibitory activities of the **10g** against ROS1, c-Src, Lyn, IRK (insulin receptor kinase) and c-Met.

The inhibitory activities of **10g** against ROS1, c-Met, IRK (insulin receptor kinase), c-Src, and Lyn were assessed by estimating IC_50_ values derived from biochemical kinase assay (Figure 2). As is expected by considering its structural similarity to entrectinib, **10g** inhibits ROS1 (IC_50_ < 1 nM) as well as ALK-wt (IC_50_ < 0.5 nM). As a matter of fact, **10g** is the most extremely potent inhibitor of ROS1 (IC_50_ < 1 nM). The activity of **10g** against c-Met is low (IC_50_ = 3775 nM) and it is over 7000-fold more potent against ALK-wt (IC_50_ < 0.5 nM) than c-Met. This observation indicates that **10g** possesses extremely high selectivity over c-Met. It is notable that crizotinb inhibits c-Met as well as it does ALK with IC_50_ values less than 1 nM^23^. Therefore, **10g** is superior to crizotinib in terms of its selectivity over c-Met. IRK is much less inhibited (IC_50_ = 404 nM) by **10g** than is ALK, which could be significantly advantageous in terms of toxicity because the alteration of the insulin receptor kinase activity could causes insulin resistance^45^. It is of interest to point out that **10g** significantly inhibits both c-Src (IC_50_ = 7 nM) and Lyn (IC_50_ = 33 nM), a Src family kinase, because it was reported that Lyn^46^ regulates activation of EGFR in lung cancer cells and c-Src^47^ is a potential therapeutic target in alectinib-resistant patients. Therefore, it is anticipated that the remarkable activities of **10g** against both c-Src and Lyn contribute to its potential as lead for lung cancer chemotherapy. We next measured the kinase-inhibitory activities of **10g** against other clinically relevant ALK mutants including DFG motif mutant F1174L, aC helix mutant C1156Y, T1151-L1152insT. As shown in Table 5, it was observed that **10g** strongly inhibits all of these mutants with single-digit nanomolar IC_50_ values.

**Table 5.** Kinase-inhibitory activities of **10g** against clinically relevant ALK mutants.

Enzyme	IC_50_ (nM)^a^
	**10g**
ALK-wt	<0.5
ALK-L1196M	<0.5
ALK-C1156Y	1.81
ALK-F1174L	8.17
ALK-T1151-L1152insT	6.19

^a^Radiometric biochemical kinase assay.

### Inhibition by 10g of ALK signalling in ALK-driven cell lines

**Figure 3.**
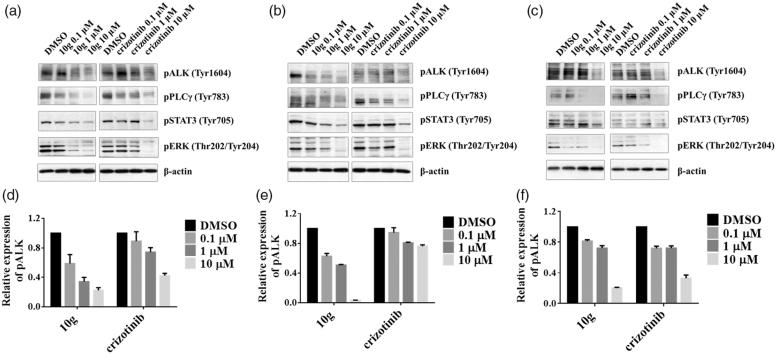
Autophosphorylation of ALK and its downstream signalling are inhibited by **10g**. (a) effects of **10g** in ALK wt-TEL Ba/F3 (b) ALK L1196M-TEL Ba/F3 (c) H2228 cell line. Ba/F3 cells transformed with ALK wt-TEL, ALK L1196M-TEL and H2228 cells were treated for 2 h with increasing concentration of **10g**.

In order to elucidate whether **10g** is capable of decreasing the level of ALK phosphorylation and deactivating downstream signalling molecules in a cellular context, western blot analysis was carried out using H2228 and Ba/F3 cells transformed with ALK-wt/ALK-L1196M. In agreement with the results of biochemical kinase assays and antiproliferative activity assays, **10g** (1 and 10 µM) effectively attenuates ALK autophosphorylation in ALK wt-TEL Ba/F3, ALK L1196M-TEL Ba/F3 and H2228 cell lines (Figure 3). Also, we found that the phosphorylation levels of STAT3, ERK and PLC-gamma, which are ALK downstream signalling molecules, are moderately suppressed by **10g** in a dose-dependent manner. Consistent with the results of biochemical kinase assays and antiproliferative activity assays, **10g** is more effective than crizotinib in inhibiting ALK autophosphorylation of ALK L1196M-TEL Ba/F3 cells. These results indicate that **10g** effectively inhibits the gatekeeper mutant ALK-L1196M as well as ALK-wt in a cellular context.

### Effects of 10g on apoptosis and cell cycle arrest in ALK-driven cell lines

**Figure 4.**
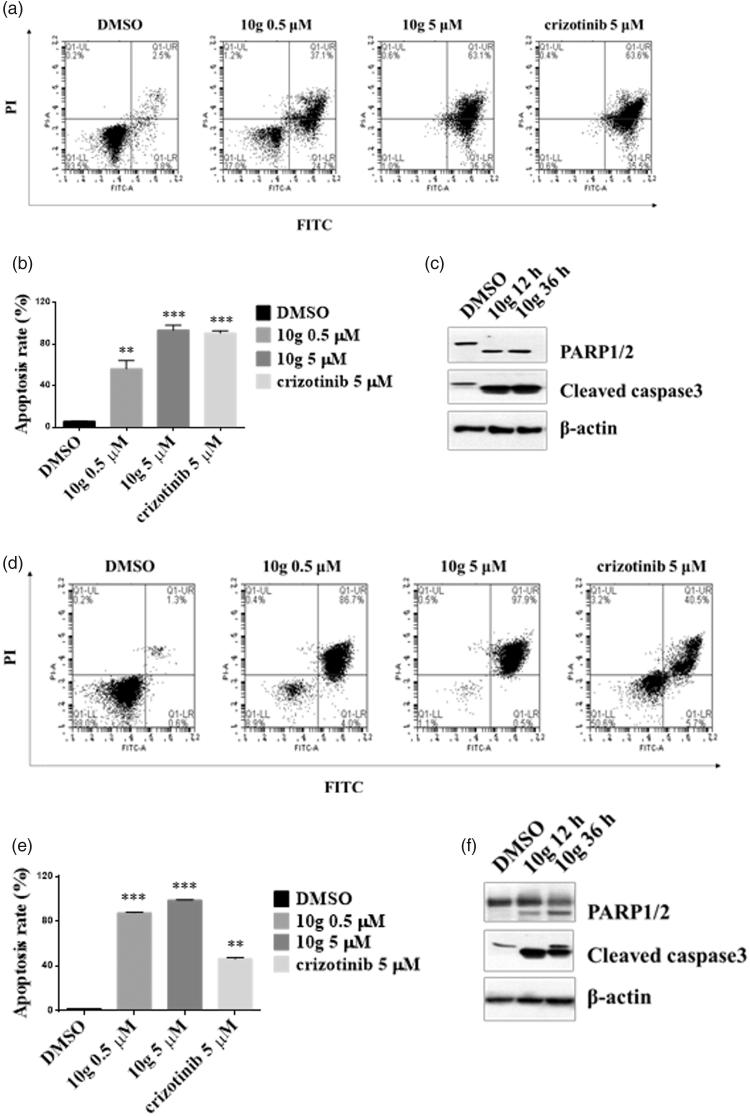
**10g** induced apoptosis in Ba/F3 cells transformed with ALK-TEL. These cell lines were incubated with 24 h and 48 h, respectively. (a, b) ALK wt-TEL Ba/F3 (d, e) ALK L1196M-TEL Ba/F3 cells were harvested and incubated in a binding buffer containing propidium iodide and annexin V-FITC and then analysed by FACS. *t*-test **p < .05, ***p < .005. Treatment with **10g** for 36 h increased cleaved PARP1/2 and cleaved caspase-3 in Ba/F3 cell transformed with (c) ALK wt-TEL and (f) ALK L1196M-TEL Ba/F3 cells.

**Figure 5.**
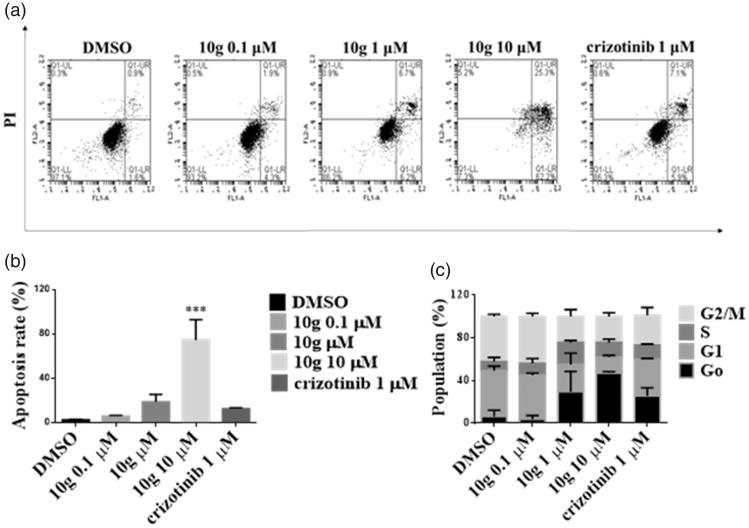
Effect on the cell apoptosis and cell cycle arrest in H2228 cells. (a, b) Percent of apoptotic cells detected by FACS analysis in H2228 cells. cells were harvested and incubated in a binding buffer containing propidium iodide and annexin V-FITC. *t*-test ***p < .005. (c) H2228 cells were incubated with indicated concentration of 10 g, fixed and stained with propidium iodide and analysed by using FACS.

Apoptosis plays a pivotal role in anticancer therapy and several ALK inhibitors are able to induce cancer cell apoptosis. Therefore, we determined whether **10g** is capable of inducing apoptosis in Ba/F3 cells transformed with ALK-TEL. Annexin V-FITC and PI staining was performed to estimate the rate of formation of apoptotic cells after **10g** treatment in Ba/F3 cells transformed with ALK wt-TEL and ALK L1196M-TEL. FACS analysis showed that treatment with **10g** for 24 h increases the number of apoptotic cells in a dose-dependent manner (Figure 4(a,b,d,e)). Furthermore, **10g** induces apoptosis of ALK L1196M-TEL Ba/F3 cells more effectively than does crizotinib (Figure 4(d,e)). In order to confirm that **10g** induces apoptosis of Ba/F3 cells transformed with ALK wt-TEL and ALK L1196M-TEL, the presence of apoptosis related protein markers was assessed using western blotting. Treatment with **10g** results in increases in the levels of cleaved PARP and cleaved caspase-3 (Figure 4(c,f)). The results demonstrate that the antiproliferative activity of **10g** against ALK-driven Ba/F3 cells is associated with its apoptosis induction capability. The effects of **10g** on apoptosis and cell cycle arrest in H2228 NSCLC cells harbouring EML4-ALK were also determined. FACS analysis revealed that treatment with **10g** for 48 h induces apoptosis in H2228 cells in a dose dependent fashion (Figure 5(a,b)). Also, treatment with 1 µM **10g** for 24 h leads to a significant enhancement of G1-S arrest in H2228 cells (Figure 5(c)), suggesting that **10g** inhibits cell proliferation via apoptosis and cell cycle arrest.

### Molecular docking studies of 10g with ALK-wt and ALK-L1196M

**Figure 6.**
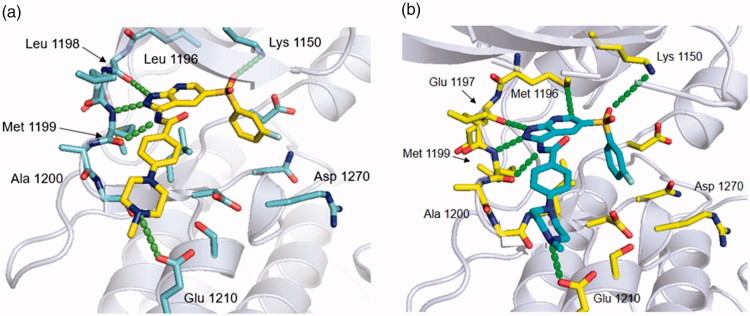
Docking study of **10g** on ALK kinase domain (a) WT and (b) L1196M based on an X-ray crystal structure (PDB code: 2XP2).

**Figure 7.**
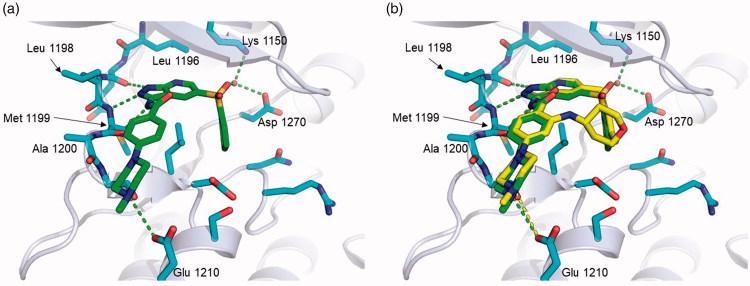
The predicted binding mode (a) of **10g** (carbon in green) based on an X-ray crystal structure (PDB code: 5FTO) is superimposed (b) to the X-ray binding mode of entrectinib (carbon in yellow).

To better understand the high kinase-inhibitory activity of **10g** on ALK, molecular docking studies were carried out using the X-ray co-crystal structures of the kinase domain of ALK complexed with crizotinib (PDB code: 2XP2) and entrectinib (PDB code: 5FTO) using Glide (Figures 6 and 7). Analysis of the results reveals that **10g** forms three hydrogen bonds with the backbone carbonyl oxygen of E1197 and backbone NH-carbonyl group of M1199 in the hinge region. It is worthwhile to note that **10g** is capable of engaging in a favourable interaction with M1196 in the kinase domain of ALK-L1196M (Figure 6(b)). It is important to note that M1196 sterically clashes with crizotinib and creates unfavourable interactions with amino group and methyl substituents of crizotinib^28^. Moreover, the sulfone group of **10g** participates in H-bonding with K1150 in the kinase domain of ALK-L1196M, which might be the reason for the improved binding affinity of **10g** with both wt and L1196M. In contrast to crizotinib, **10g** is involved in a H-bonding interaction with E1210 in solvent exposed region, which might also contribute to extremely high potency of **10g** on ALK enzyme. In addition, the predicted binding mode of **10g** was superimposed to the X-ray binding mode of entrectinib (Figure 7). This superimposition reveals that the sulfone linker in **10g** makes a water molecule-mediated hydrogen bond with K1150 and D1270 while the methylene linker of entrectinib is not capable of forming this H-bond.

## Conclusions

In current effort, we designed and synthesised novel 3-amino-5-substituted pyrozolopyridine derivatives and assessed their kinase-inhibitory activities against ALK-L1196M gatekeeper mutant as well as against ALK-wt, and their antiproliferative activities on Ba/F3 cells transformed with ALK-wt/ALK-L1196M and on H2228 non-small cell lung cancer cells harbouring EML4-ALK. The pyrozolopyridine derivative **10g** was found to have exceptional kinase-inhibitory activities against both ALK-L1196M (IC_50_ < 0.5 nM) and ALK-wt (IC_50_ < 0.5 nM). It was reported that entrectinib^11^^,^^38^ inhibits ALK-wt with IC_50_ value of 12 nM and crizotinib inhibits ALK-L1196M with IC_50_ value of 980 nM. Moreover, **10g** is extremely potent against ROS1 (IC_50_ < 0.5 nM) and it possesses a high selectivity (>7000 fold) over c-Met. Meanwhile, the high activities of **10g** on both c-Src (IC_50_ = 7 nM) and Lyn (IC_50_ = 33 nM) could contribute to its potential as a novel lead for lung cancer treatment. Also, **10g** strongly suppresses the proliferation of both H2228 cells and ALK-driven Ba/F3 cells. It should be emphasised that **10g** more profoundly (GI_50_ = 0.129 µM) blocks proliferation of ALK-L1196M Ba/F3 cells than does crizotinib (GI_50_ = 0.726 µM). Moreover, **10g** is 27-fold more potent against ALK-L1196M Ba/F3 cells than on parental Ba/F3 cells and, in comparison to crizotinib, it exhibits a much more favourable differential cytotoxicity. The results of western blot analysis reveal that **10g** dose-dependently attenuates phosphorylation of ALK downstream signalling molecules (STAT3, ERK and PLC-gamma) as well as ALK autophosphorylation in ALK wt-TEL Ba/F3, ALK L1196M-TEL Ba/F3 and H2228 cell lines. Also, **10g** inhibits ALK autophosphorylation on ALK L1196M-TEL Ba/F3 cells more potently than does crizotinib. The results also show that **10g** exerts its antiproliferative effect by inducing apoptosis, as evidenced by the fact that it markedly induces apoptotic markers (cleaved PARP and cleaved caspase 3) on H2228 cells as well as on ALK-driven Ba/F3 cells. The results of docking study of **10g** on ALK-wt/ALK-L1196M kinase domains demonstrate that **10g** engages in three hydrogen bonds with backbone E1197 and M1199 in the hinge region. In contrast to crizotinib, **10g** participates in favourable interactions with M1196 in the kinase domain of ALK-L1196M and two additional hydrogen bonds with K1150 and E1210, which likely contributes to its exceptional potency against ALK enzyme. The investigation described above has provided insight into new strategies to design novel and potent ALK-L1196M inhibitors that circumvent crizotinib resistance.
